# Effect of Chewing Gum on Postoperative Gastrointestinal Recovery in Patients Undergoing Abdominal Surgery: A Randomized Controlled Study

**DOI:** 10.7759/cureus.106979

**Published:** 2026-04-13

**Authors:** Anil Kumar LN, Sudeep Saran, Rajeev Ranjan

**Affiliations:** 1 Department of Anaesthesia, Seth Gordhandas Sunderdas Medical College and King Edward Memorial (KEM) Hospital, Mumbai, IND; 2 Department of Diabetologists and Critical Care, Saran Hospital and Institute of Paramedical Sciences, Bareilly, IND; 3 Department of General Surgery, Netaji Subhas Medical College and Hospital, Bihta, IND

**Keywords:** abdominal surgery, chewing gum, enhanced recovery after surgery, gastrointestinal recovery, postoperative ileus

## Abstract

Background

Postoperative ileus is a common complication following abdominal surgeries, leading to delayed recovery, prolonged hospital stay, and increased patient discomfort. Chewing gum, a form of sham feeding, has been proposed as a simple, non-pharmacological intervention to stimulate gastrointestinal motility and hasten postoperative recovery. This study aimed to evaluate the effect of chewing gum on postoperative recovery of gastrointestinal function in patients undergoing abdominal surgery.

Methods

This single-blinded randomized controlled study included 198 adult patients undergoing elective abdominal surgeries, who were randomly allocated to a chewing gum group (n = 99) or a control group (n = 99). Patients in the intervention group received postoperative chewing gum once fully conscious with intact airway reflexes, while the control group received standard postoperative care. Primary outcomes included the time to passage of first flatus and the appearance of first bowel sound. Secondary outcomes were postoperative nausea and vomiting (PONV), length of hospital stay, and safety outcomes. Time-to-event analyses were performed using Kaplan-Meier curves with log-rank tests, and multivariable logistic regression was used to identify independent predictors of gastrointestinal recovery.

Results

Baseline demographic and surgical characteristics were comparable between groups. The chewing gum group demonstrated significantly earlier passage of first flatus (197.0 ± 3.6 vs 280.0 ± 6.3 minutes; p < 0.0001) and earlier appearance of first bowel sounds (201.5 ± 3.8 vs 280.0 ± 6.3 minutes; p < 0.0001). These benefits were consistent across surgical approaches and gender subgroups. The incidence and duration of PONV were significantly lower in the chewing gum group, with faster complete remission (126.0 ± 13.5 vs 237.5 ± 13.1 minutes; p < 0.0001). The hospital stay was significantly shorter in the chewing gum group (1.7 ± 1.3 vs 3.1 ± 1.7 days; p < 0.001). On multivariable analysis, chewing gum independently predicted earlier gastrointestinal recovery (OR = 0.29, 95% CI 0.15-0.59; p < 0.001). No intervention-related adverse events were observed.

Conclusion

Chewing gum significantly accelerates postoperative gastrointestinal recovery, reduces PONV, and shortens hospital stay following abdominal surgery. Given its safety, simplicity, and low cost, chewing gum should be considered a valuable adjunct to standard postoperative care and Enhanced Recovery After Surgery protocols.

## Introduction

Postoperative ileus (POI) represents a frequent and clinically important sequela of abdominal surgery, resulting from a temporary disruption of coordinated gastrointestinal (GI) motility and a consequent delay in the return of normal bowel function. Reported incidence rates range from 15 to 30% following major abdominal operations and may rise to nearly 40% after colorectal procedures, where extensive bowel handling is common [1,2]. POI imposes a substantial burden on postoperative outcomes, contributing to 12-15% of unexpected hospital readmissions and extending the duration of hospitalization by approximately 1.5-5 days. This delay translates into increased healthcare expenditure, estimated at $1,200-$4,000 per affected patient [3,4]. From a clinical standpoint, POI is characterized by abdominal bloating, delayed passage of flatus or stool, nausea, vomiting, and poor tolerance of oral intake, all of which impede early mobilization and prolong postoperative recovery.

The development of POI is driven by multiple interacting mechanisms, including surgery-related sympathetic nervous system activation, local and systemic inflammatory responses with the release of mediators such as interleukin-6 and tumor necrosis factor-α, opioid-induced suppression of intestinal motility, and transient dysfunction of the enteric nervous system [5]. Although minimally invasive surgical approaches and advances in anesthetic and perioperative management have reduced overall surgical stress, POI continues to occur at clinically relevant rates. Contemporary Enhanced Recovery After Surgery (ERAS) protocols advocate strategies such as early oral feeding, opioid-sparing analgesia, early ambulation, and optimized perioperative fluid management; however, adherence to these recommendations and their effectiveness remain inconsistent across healthcare settings [6]. This variability underscores the need for additional, low-risk interventions that are easy to implement and broadly applicable.

Chewing gum has been proposed as a simple, non-pharmacological adjunct to enhance postoperative bowel recovery through the principle of sham feeding. Mastication activates cephalic-vagal reflexes, leading to increased salivary and gastrointestinal secretions and the stimulation of gut-related hormones, including gastrin and neurotensin, which collectively promote intestinal peristalsis [7]. Experimental and clinical studies have demonstrated that chewing gum can augment vagal tone by 10-20% and facilitate gastric emptying and colonic transit [8]. Randomized trials and meta-analyses have reported clinically meaningful benefits, including reductions of 6-24 hours in time to first flatus, 12-36 hours in time to first bowel movement, shorter hospital stay by 0.5-1.5 days, and a 22-30% reduction in the risk of prolonged ileus [9-11]. Nevertheless, differences in study populations, surgical procedures, outcome definitions, and chewing protocols limit the generalizability of the existing evidence.

In this context, this randomized controlled study was designed to evaluate the effect of postoperative chewing gum on gastrointestinal recovery in patients undergoing abdominal surgery, with a specific assessment of clinically relevant recovery outcomes.

## Materials and methods

Study design and setting

This single-blinded randomized controlled study was conducted in the Department of Anaesthesiology in collaboration with the Department of General Surgery at a tertiary care teaching hospital for a period of one year between November 2018 and November 2019. The objective was to evaluate the effect of chewing gum on the postoperative recovery of gastrointestinal function in patients undergoing abdominal surgery. 

Study population

The study population comprised adult patients aged ≥18 years undergoing elective abdominal surgeries, including both open and laparoscopic procedures, performed under general anesthesia. Additional inclusion criteria required that patients demonstrate intact airway reflexes following extubation to ensure safe participation in the chewing intervention. Patients who refused consent, were unable to be extubated following surgery, were pregnant or lactating, or had conditions that impaired participation were excluded. Patients who required prolonged postoperative mechanical ventilation or developed immediate major postoperative complications were predefined as exclusion criteria; however, no enrolled participants met these criteria, and therefore, all randomized patients were included in the final analysis.

Sample size calculation

Sample size estimation was based on a reference study by Shang et al., which reported a mean time to first flatus of 34.6 ± 12.6 hours in the chewing gum group and 39.9 ± 13.5 hours in the control group [12]. The mean difference of 5.3 hours (SD ± 12.6) was considered clinically significant. Using a two-sided t-test with α = 0.05 and 80% power, the sample size was calculated using the formula: n = 2 × (Zα/2 + Zβ)² × (σ/d)², where Zα/2 = 1.96, Zβ = 0.84, σ = 12.6, and d = 5.3. Substituting the values resulted in an estimated requirement of 90 patients per group. Allowing for a 10% dropout rate, the final calculated sample size was 99 patients per group, totaling 198 participants. Sample size estimation was performed using Power and Sample Size Software (version 3.1.6).

Randomization, allocation, and blinding

**Figure 1:**
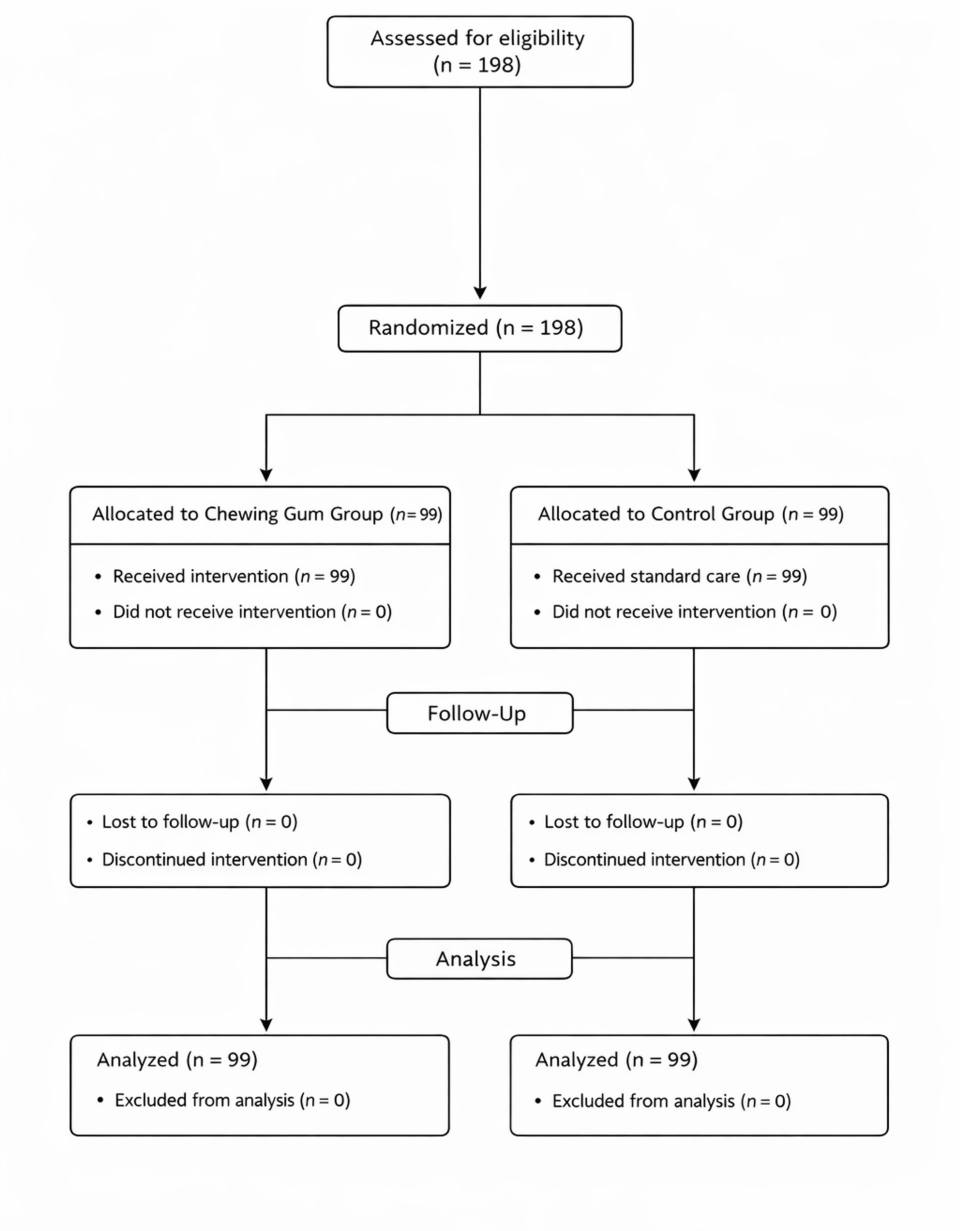
CONSORT flow diagram of participant enrollment, randomization, follow-up, and analysis.

A total of 198 eligible and consenting patients were randomized using computer-generated simple randomization into two equal groups: the chewing gum intervention group and the control group. Allocation concealment was ensured using sequentially numbered, sealed, opaque envelopes opened only after the patient had been transferred to the postoperative recovery area. Because of the nature of the intervention, blinding of participants was not feasible; however, the study maintained single blinding by ensuring that outcome assessors and data analysts were unaware of group allocation (Figure 1).

Anesthetic and analgesic protocol

All procedures were performed under standardized general anesthesia in accordance with established institutional protocols and routine perioperative practice. Induction, maintenance agents, airway management, neuromuscular blockade, intraoperative monitoring, and reversal strategies followed uniform institutional guidelines and were identical for both study groups. Epidural anesthesia and intravenous patient-controlled analgesia (IV-PCA) were not routinely employed for the abdominal procedures included in this study as part of prevailing institutional practice. Their non-use was independent of the study design and did not represent the withholding of standard care.

Postoperative analgesia was administered using a standardized multimodal regimen for all patients. Intravenous paracetamol 1 g was given every 8 hours during the first 24 hours postoperatively. In addition, intravenous diclofenac 75 mg was administered every 12 hours unless contraindicated (e.g., renal dysfunction, bleeding risk, or hypersensitivity). When diclofenac was contraindicated, intravenous ketorolac 30 mg every 8-12 hours was used as an alternative non-steroidal anti-inflammatory drug. Rescue opioid analgesia consisted primarily of intravenous tramadol 50-100 mg every 6-8 hours, as required, for breakthrough pain (visual analog scale score ≥4). If pain control remained inadequate, intravenous fentanyl 25-50 µg boluses were administered under clinical supervision. Pain was assessed at 6-hour intervals during the first postoperative day using standardized pain scoring systems, and analgesic adjustments were made accordingly to ensure adequate patient comfort.

Importantly, no analgesic modality was withheld for research purposes. All patients received pain management consistent with institutional standards of care, and anesthetic and analgesic protocols were kept consistent across both study groups to minimize potential confounding effects on postoperative gastrointestinal recovery and the incidence of postoperative nausea and vomiting.

Intervention protocol

Patients allocated to the intervention group received commercially available sugar-free chewing gum (Orbit® Sugar-Free Gum, Mars Wrigley Confectionery, USA; manufactured in India). The same brand, formulation, and flavor were used for all participants to ensure uniformity. Patients were instructed to chew one piece of gum for approximately 20 minutes per session, beginning after full recovery from anesthesia and confirmation of intact airway reflexes. Chewing was performed three times daily (morning, afternoon, and evening) under nursing supervision and continued until the passage of first flatus. Compliance was documented during hospitalization. The control group received standard postoperative care without chewing gum. All patients in both groups were monitored at 30-minute intervals after extubation for hemodynamic parameters, including temperature, heart rate, and blood pressure. Gastrointestinal recovery was assessed objectively by healthcare personnel. Auscultation for bowel sounds was performed every 30 minutes during the first six postoperative hours and subsequently at hourly intervals until bowel sounds were detected or the patient reported passage of first flatus. Time to event was recorded in minutes from extubation.

Outcome measures

The primary outcome was the time to first passage of flatus (in hours) following surgery. Secondary outcomes included time to first bowel sound, duration of postoperative hospital stay, and incidence of postoperative complications such as delayed wound healing, fever, tachycardia, leukocytosis, and postoperative nausea and vomiting (PONV). The outcome time to first bowel sound was assessed objectively by trained healthcare professionals through standardized abdominal auscultation at predefined postoperative intervals. In contrast, patient-reported outcomes included time to passage of first flatus and symptoms of postoperative nausea and vomiting, which were recorded based on patient reporting and subsequently verified by clinical staff. Postoperative complications were prospectively and systematically assessed in all enrolled patients using predefined clinical criteria. Fever >38°C, heart rate >100 beats/min, and total leukocyte count >12,000/mm³ were considered indicators of infection. Wound healing complications, including soakage, purulent discharge, or increased dressing frequency (>2 per day), were recorded. PONV severity was assessed using a predefined grading system: Grade 0, no nausea or vomiting; Grade 1, nausea without vomiting; Grade 2, nausea with <3 vomiting episodes in 24 hours; Grade 3, nausea with ≥3 episodes of vomiting in 24 hours [13]. Patients with Grade ≥2 PONV received antiemetic therapy. All adverse events were documented and appropriately managed. Delayed postoperative gastrointestinal recovery was defined a priori as failure to pass first flatus within the median time observed in the overall cohort (≥240 minutes), consistent with our time-to-event distribution. Patients exceeding this threshold were categorized as having delayed recovery (Yes), while those achieving flatus before this time were categorized as early recovery (No).

Perioperative standardization

Perioperative management was standardized across both study arms to reduce variability. Intraoperative fluid therapy followed goal-directed principles. Early postoperative mobilization was encouraged once patients were hemodynamically stable. Oral intake was initiated according to institutional protocols once bowel sounds were present and nausea was controlled, with no differences between groups. All perioperative decisions were made independently of study allocation.

Oral intake was initiated with clear liquids once patients were fully awake and hemodynamically stable, typically within 6-8 hours postoperatively, and advanced as tolerated according to institutional enhanced recovery guidelines. Early mobilization was encouraged within the first 12-24 hours after surgery under supervision. The timing of extubation was recorded for all patients, and identical postoperative care pathways were followed in both groups. No protocol deviations were allowed unless clinically indicated.

Statistical analysis

Data were entered into Microsoft Excel (Microsoft Corp., Redmond, WA, USA) and analyzed using IBM SPSS Statistics for Windows, Version 20.0 (Released 2011; IBM Corp., Armonk, NY, USA). Continuous variables such as age and recovery times were summarized as mean ± standard deviation, and 95% confidence intervals were reported where appropriate. Categorical variables, including gender, type of surgery, and incidence of postoperative nausea and vomiting, were expressed as frequencies and percentages. Time-to-event outcomes, specifically time to passage of first flatus, time to appearance of first bowel sound, and time to complete remission of postoperative nausea and vomiting, were analyzed using Kaplan-Meier survival curves, with comparisons between groups performed using the log-rank (Mantel-Cox) test. Between-group comparisons of continuous outcomes were conducted using the independent t-test. Associations between patient characteristics (gender and type of surgery) and gastrointestinal recovery outcomes were further explored using stratified analyses and general linear models where appropriate. Categorical outcomes were analyzed using the chi-square test or Fisher’s exact test as applicable. Multivariable binary logistic regression analysis was performed to identify independent predictors of delayed postoperative gastrointestinal recovery. A p-value of <0.05 was considered statistically significant for all analyses.

Ethical statement

The trial (CTRI registration number: CTRI/2018/11/016427) adhered to the principles of the Declaration of Helsinki. Institutional Ethics Committee approval (Seth GS Medical College and KEM Hospital, Mumbai, Maharashtra, IEC/104/2018; dated 15 October 2018) was obtained before commencement of the study, and written informed consent in the language best understood by each participant was secured. A copy of the consent form was provided to all enrolled patients.

## Results

A total of 198 patients were enrolled and randomized in the study, with 99 patients allocated to each group. None of the enrolled participants required prolonged postoperative mechanical ventilation or experienced immediate postoperative complications necessitating exclusion from analysis. Therefore, no patients were excluded after randomization, and all randomized participants were included in the final analysis. The mean age of participants was comparable between the chewing gum group (42.5 ± 14.6 years) and the control group (43.1 ± 14.8 years), with no statistically significant difference (p = 0.772). Age-group distribution was similar across both groups, and no significant difference was observed (p = 0.687). Gender distribution was balanced, with males comprising 33.3% in the chewing gum group and 42.4% in the control group (p = 0.187). Laparoscopic procedures were the predominant surgical approach in both groups (77.8% vs 69.7%), while open surgeries constituted 22.2% and 30.3% of cases in the chewing gum and control groups, respectively; this difference was not statistically significant (p = 0.196). The spectrum of open and laparoscopic procedures was comparable between the two groups, with cholecystectomy being the most common laparoscopic surgery and Frey’s procedure and Whipple’s procedure among the more frequent open surgeries. No postoperative complications such as tachycardia, delayed wound healing, or postoperative infections were observed in either group during the study period (Table 1).

**Table 1:** Baseline demographic, clinical characteristics, and surgical profile of study participants. CBD: common bile duct; TARM: transversus abdominis muscle release.

Variable	Subvariables	Chewing gum (n = 99)	Control (n = 99)	Test statistic, p value
		Frequency (%)/mean ± SD		
Age (years)		42.5 ± 14.6	43.1 ± 14.8	t = −0.29 , 0.772
Age group (years)	18–20	8 (8.1)	4 (4.0)	χ² = 3.03 , 0.687
	21–30	16 (16.2)	18 (18.2)	
	31–40	24 (24.2)	31 (31.3)	
	41–50	21 (21.2)	16 (16.2)	
	51–60	15 (15.2)	16 (16.2)	
	>60	15 (15.2)	14 (14.1)	
Gender	Male	33 (33.3)	42 (42.4)	χ² = 1.74, 0.187
	Female	66 (66.7)	57 (57.6)	
Type of surgery (overall)	Open	22 (22.2)	30 (30.3)	χ² = 1.67, 0.196
	Laparoscopic	77 (77.8)	69 (69.7)	
Open surgeries	Frey’s procedure	3 (13.6)	6 (20.0)	_
	Hepaticojejunostomy	4 (18.2)	2 (6.7)	_
	Appendectomy	2 (9.1)	0 (0.0)	_
	CBD exploration	1 (4.5)	0 (0.0)	_
	Colectomy	2 (9.1)	2 (6.7)	_
	Gastrectomy	1 (4.5)	1 (3.3)	_
	Gastrojejunostomy	1 (4.5)	0 (0.0)	_
	Hemicolectomy	1 (4.5)	2 (6.7)	_
	Pancreaticojejunostomy	2 (9.1)	1 (3.3)	_
	Right hemicolectomy	2 (9.1)	4 (13.3)	_
	Splenectomy	1 (4.5)	1 (3.3)	_
	Whipple’s procedure	1 (4.5)	7 (23.3)	_
	TARM with stoma closure	1 (4.5)	0 (0.0)	_
	Exploratory laparotomy + ileal stoma	0 (0.0)	1 (3.3)	_
	Open pancreatic tumor excision	0 (0.0)	1 (3.3)	_
	Splenorenal shunt	0 (0.0)	1 (3.3)	_
	TARM	0 (0.0)	1 (3.3)	_
Laparoscopic surgeries	Cholecystectomy	59 (76.6)	51 (73.9)	_
	Umbilical hernia repair	6 (7.8)	5 (7.2)	_
	Inguinal hernia repair	5 (6.5)	7 (10.1)	_
	Heller’s myotomy	2 (2.6)	1 (1.4)	_
	Appendectomy	1 (1.3)	3 (4.3)	_
	Paraduodenal hernia repair	1 (1.3)	0 (0.0)	_
	Paraumbilical hernioplasty	1 (1.3)	0 (0.0)	_
	Retroperitoneal mass excision	1 (1.3)	0 (0.0)	_
	Splenectomy	1 (1.3)	0 (0.0)	_
	Spigelian hernia repair	0 (0.0)	1 (1.4)	_
	Ventral hernia repair	0 (0.0)	1 (1.4)	_
Postoperative complications	Tachycardia	0 (0.0)	0 (0.0)	_
	Delayed wound healing	0 (0.0)	0 (0.0)	_
	Postoperative infections	0 (0.0)	0 (0.0)	_

Patients in the chewing gum group demonstrated significantly faster recovery of gastrointestinal function compared with the control group. The mean time to passage of first flatus was significantly shorter in the chewing gum group (197.0 ± 3.6 minutes) than in the control group (280.0 ± 6.3 minutes; p < 0.0001). This difference remained statistically significant across both open and laparoscopic surgeries, as well as across gender subgroups (all p < 0.0001). Similarly, the mean time to appearance of first bowel sound was significantly reduced in the chewing gum group (201.5 ± 3.8 minutes) compared with the control group (280.0 ± 6.3 minutes; p < 0.0001). Stratified analysis by type of surgery and gender consistently demonstrated earlier bowel sound recovery among patients receiving chewing gum (p < 0.0001 for all comparisons). The mean duration of postoperative hospital stay was significantly shorter in the chewing gum group (1.7 ± 1.3 days) compared with the control group (3.1 ± 1.7 days; p < 0.001). The incidence of postoperative nausea and vomiting (PONV) was markedly lower in the chewing gum group at multiple postoperative time points up to 270 minutes, with statistically significant differences observed from 30 minutes to 270 minutes postoperatively. No PONV episodes were observed beyond 210 minutes in the chewing gum group, whereas symptoms persisted up to 300 minutes in the control group. The time to complete remission of PONV was significantly shorter in the chewing gum group (126.0 ± 13.5 minutes) compared with the control group (237.5 ± 13.1 minutes; p < 0.0001) (Table 2).

**Table 2:** Comparison of postoperative gastrointestinal recovery and related outcomes between chewing gum and control groups. PONV: postoperative nausea and vomiting.

Variables	Chewing gum (n = 99)	Control (n = 99)	Test statistic, p value
	Frequency (%)/mean ± SD		
Time to pass first flatus (minutes)	197.0 ± 3.6	280.0 ± 6.3	t = −113.2, <0.0001
Type of surgery			
Open (n = 52)	256.4 ± 7.0	372.0 ± 4.5	t = −78.6, <0.0001
Laparoscopic (n = 146)	180.0 ± 2.2	240.0 ± 0.0	t = −152.4, <0.0001
Gender			
Male (n = 75)	196.4 ± 6.0	301.4 ± 10.9	t = −76.1, <0.0001
Female (n = 123)	197.3 ± 4.4	264.2 ± 7.0	t = −96.8, <0.0001
Time to appearance of first bowel sound (minutes)	201.5 ± 3.8	280.0 ± 6.3	t = −98.5, <0.0001
Type of surgery			
Open (n = 52)	256.4 ± 7.0	372.0 ± 4.5	t = −78.6, <0.0001
Laparoscopic (n = 146)	185.8 ± 2.2	240.0 ± 0.0	t = −152.4, <0.0001
Gender			
Male (n = 75)	203.6 ± 6.2	301.4 ± 10.9	t = −76.1, <0.0001
Female (n = 123)	200.5 ± 4.7	264.2 ± 6.7	t = −96.8, <0.0001
Hospital stay (days)	1.7 ± 1.3	3.1 ± 1.7	t = −6.66, <0.001
Incidence of PONV			
30 minutes	15 (15.5)	36 (36.4)	χ² = 11.1 , 0.001
60 minutes	13 (13.3)	34 (34.4)	χ² = 12.4 , 0.001
90 minutes	10 (10.1)	33 (33.3)	χ² = 14.5 , 0.001
120 minutes	5 (5.5)	33 (33.3)	χ² = 24.6 , 0.001
150 minutes	4 (4.4)	31 (31.1)	χ² = 25.9 , 0.001
180 minutes	2 (2.2)	28 (28.9)	χ² = 26.8 , 0.001
210 minutes	0 (0.0)	21 (21.1)	χ² = 24.9, 0.001
240 minutes	0 (0.0)	19 (19.9)	χ² = 22.0 , <0.0001
270 minutes	0 (0.0)	13 (18.0)	χ² = 13.9, 0.01
300 minutes	0 (0.0)	9 (15.0)	χ² = 1.39, 0.237
330 minutes	0 (0.0)	0 (0.0)	_
360 minutes	0 (0.0)	0 (0.0)	_
420 minutes	0 (0.0)	0 (0.0)	_
480 minutes	0 (0.0)	0 (0.0)	_
Time to complete remission of PONV (minutes)	126.0 ± 13.5	237.5 ± 13.1	t = −59.2, <0.0001

**Figure 2:**
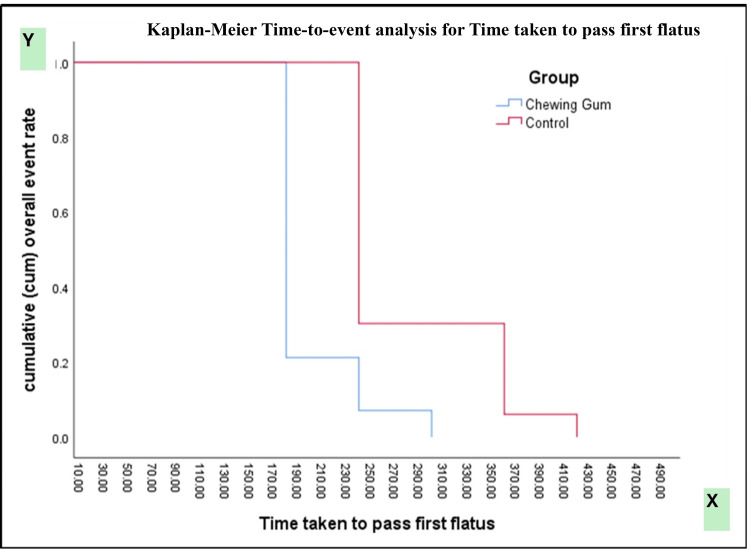
Kaplan-Meier curve for time taken to pass first flatus.

**Figure 3:**
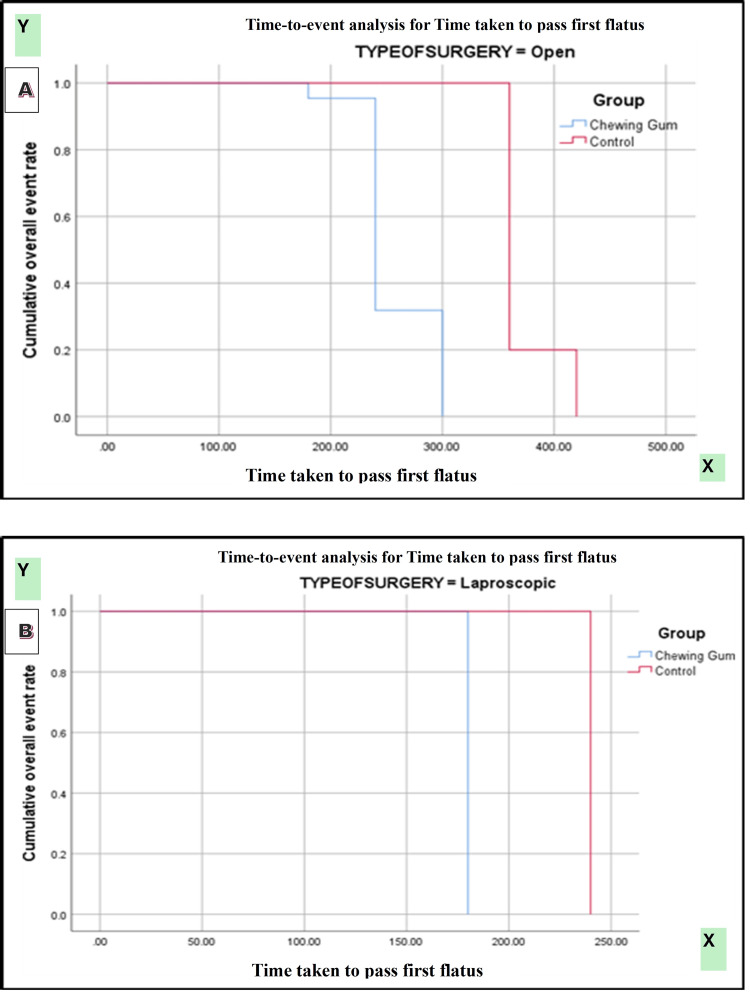
(A, B) Kaplan-Meier curve for time taken to pass first flatus between two groups according to type of surgery.

**Figure 4:**
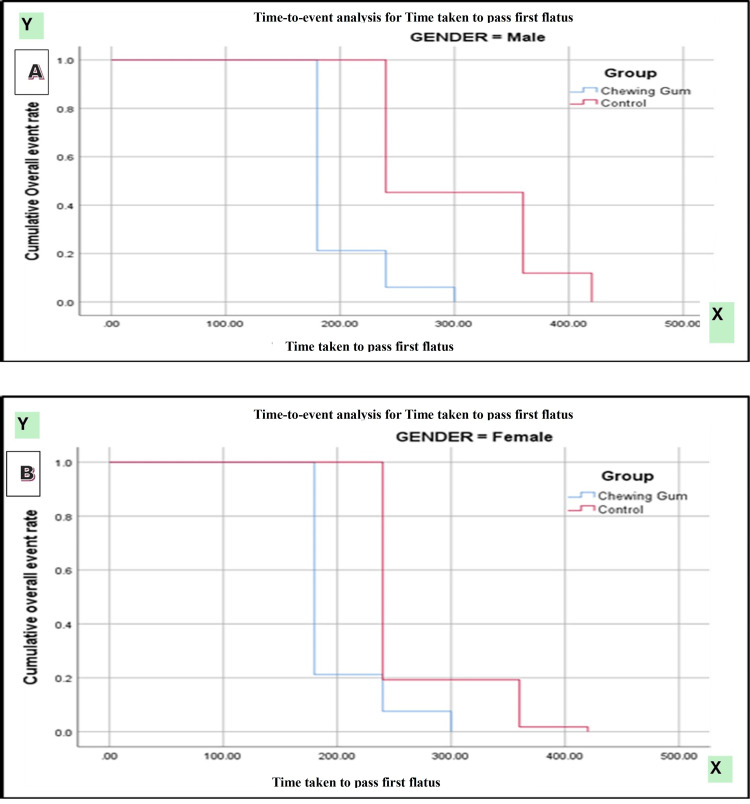
(A, B) Kaplan-Meier curve for time taken to pass first flatus according to gender.

Kaplan-Meier survival analysis demonstrated a significantly earlier passage of first flatus in patients receiving chewing gum compared with controls (log-rank [Mantel-Cox] χ² = 118.147, p < 0.0001). Subgroup analysis by type of surgery showed significantly faster recovery in both open and laparoscopic procedures among the chewing gum group (χ² = 202.534, p < 0.0001). Gender-based analysis also revealed a consistent benefit of chewing gum in both male and female patients (χ² = 114.390, p < 0.0001) (Figures 2-4).

**Figure 5:**
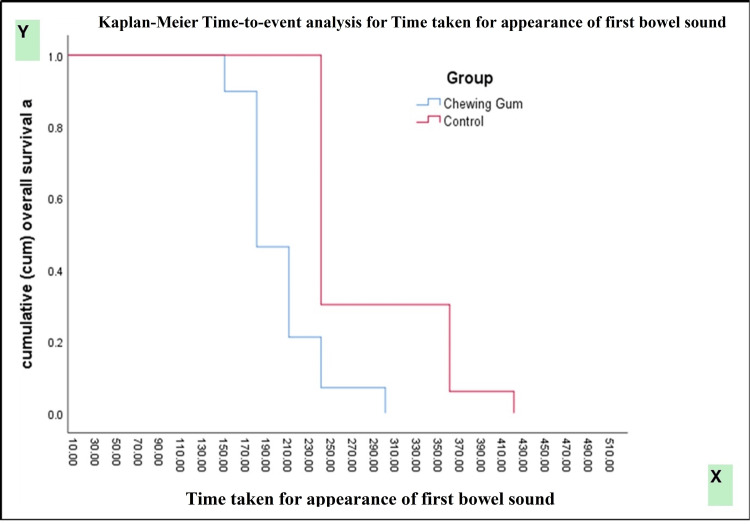
Kaplan-Meier curve for time taken for the first bowel sound.

**Figure 6:**
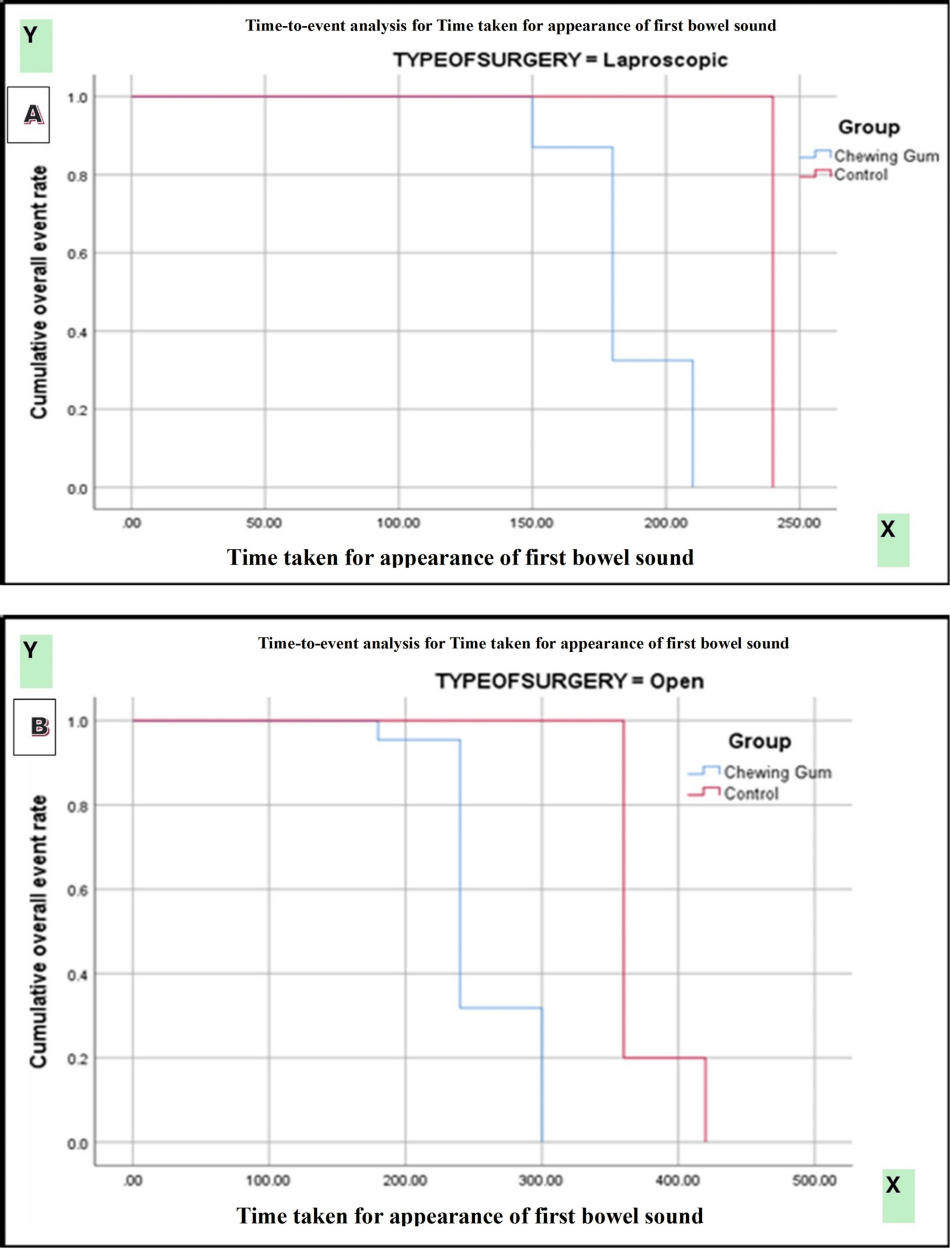
(A, B) Kaplan-Meier curve for time taken for the first bowel sound according to type of surgery.

**Figure 7:**
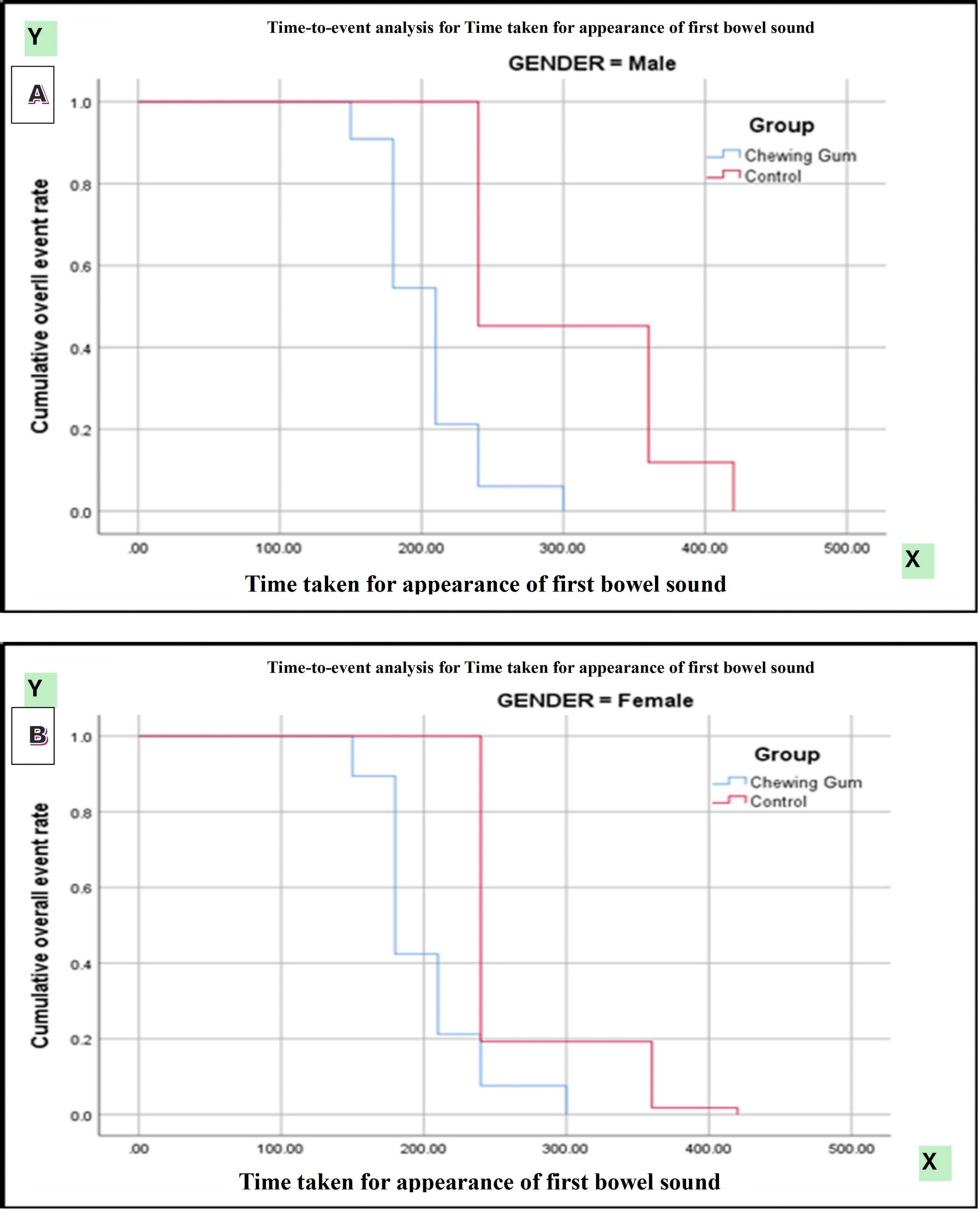
(A,B) Kaplan-Meier curve for time taken for first bowel sound according to gender.

Kaplan-Meier curves demonstrated a significantly shorter time to appearance of first bowel sound in the chewing gum group compared with the control group (log-rank χ² = 125.377, p < 0.0001). Stratified analyses showed significant differences favoring the chewing gum group across gender (χ² = 121.114, p < 0.0001) and type of surgery (χ² = 204.894, p < 0.0001) (Figures 5-7).

**Figure 8:**
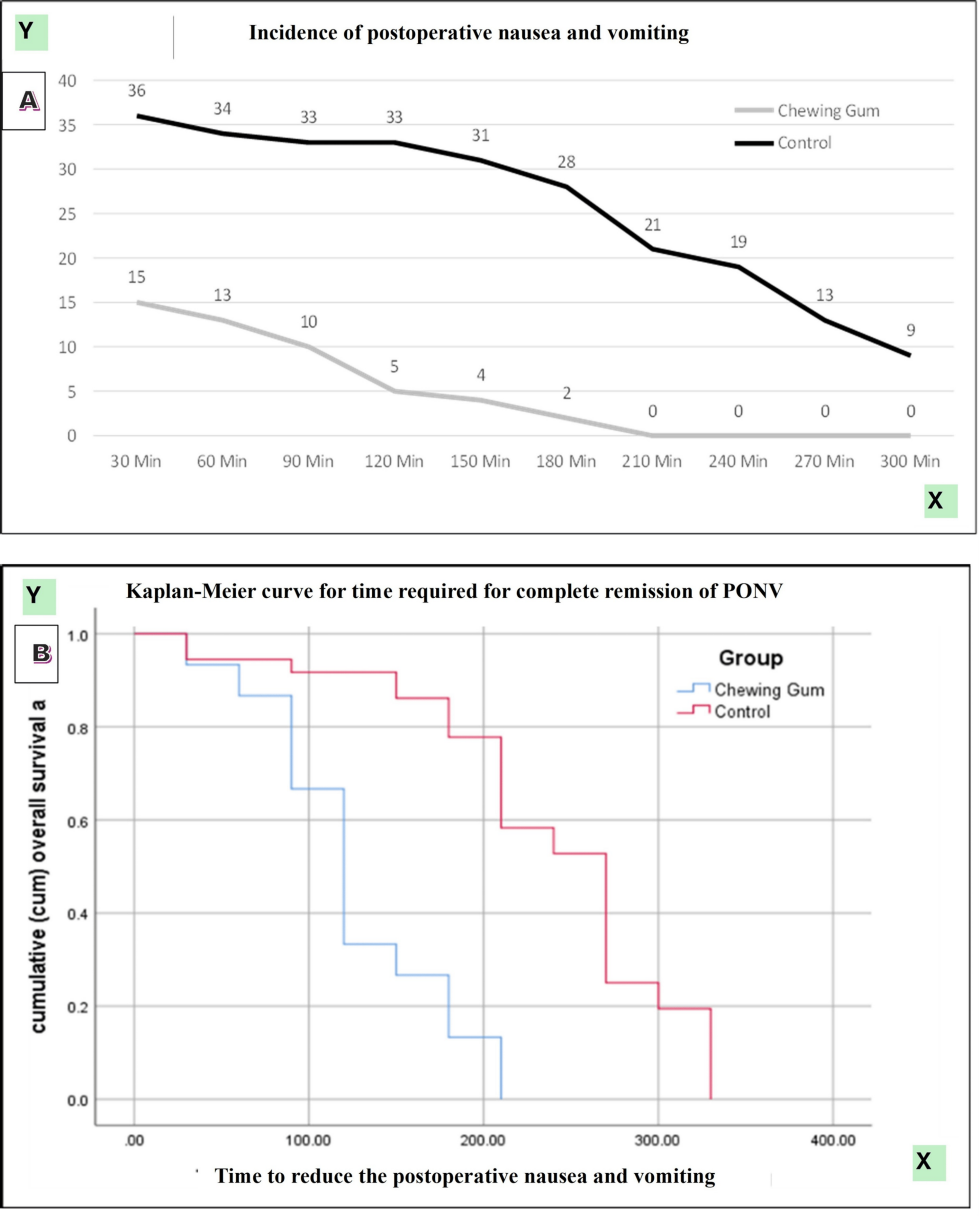
(A) Incidence of PONV. (B) Kaplan-Meier curve for time taken for complete remission of PONV. PONV: postoperative nausea and vomiting.

The incidence of postoperative nausea and vomiting was consistently lower in the chewing gum group across all observed postoperative time points. Kaplan-Meier analysis for time to complete remission of PONV demonstrated significantly faster symptom resolution in the chewing gum group compared with controls (log-rank χ² = 28.224, p < 0.0001) (Figure 8).

Multivariable logistic regression analysis was performed to identify independent predictors of delayed postoperative gastrointestinal recovery. After adjusting for age, gender, and surgical approach, chewing gum remained a significant independent predictor of earlier recovery. Patients in the control group had significantly lower odds of early gastrointestinal recovery compared with those in the chewing gum group (adjusted OR = 0.31, 95% CI 0.15-0.62; p < 0.001). Age, gender, and surgical approach (open versus laparoscopic) were not independently associated with delayed recovery (Table 3).

**Table 3:** Multivariable logistic regression analysis of factors associated with delayed postoperative gastrointestinal recovery. CI: confidence interval; OR: odds ratio.

Variables	Category	Adjusted OR (Exp B)	95% Confidence interval	p value
Age (years)	Continuous	0.98	0.96–1.01	0.17
Gender	Female	0.72	0.35–1.47	0.36
	Male (reference)	1	—	—
Surgical approach	Open	1.41	0.69–2.88	0.34
	Laparoscopic (Reference)	1	—	—
Study group	Control	0.31	0.15–0.62	<0.001
	Chewing gum (Reference)	1	—	—

## Discussion

Delayed recovery of gastrointestinal function continues to pose a significant challenge after abdominal surgery, contributing to patient discomfort, prolonged hospitalization, and increased healthcare expenditure. In the present randomized controlled study, postoperative chewing gum was associated with a markedly faster return of bowel function. The most notable finding was the significantly earlier passage of first flatus in patients who chewed gum compared with those who received standard care alone (197.0 ± 3.6 vs 280.0 ± 6.3 minutes; p < 0.0001). This nearly one-third reduction in recovery time is clinically relevant, as delayed flatus is widely regarded as a key marker of postoperative ileus. Comparable benefits have been documented in earlier meta-analyses and randomized trials, including those by Li et al., Vergara-Fernandez et al., Song et al., and Mei et al., which reported reductions ranging from 6 to 24 hours in postoperative bowel recovery following gum chewing [14-17]. Likewise, Shang et al. observed a mean difference of approximately 5.3 hours in time to first flatus, closely mirroring the magnitude of effect demonstrated in our cohort [12].

The beneficial impact of chewing gum is commonly attributed to its role as a form of sham feeding. Mastication initiates cephalic-phase responses that stimulate vagal efferent pathways, resulting in increased salivary output, gastric and pancreatic secretion, and release of gut-related hormones such as gastrin and neurotensin [18,19]. These neurohumoral responses promote intestinal peristalsis without exposing patients to the risks of early oral intake [20]. Experimental evidence also suggests that chewing gum enhances parasympathetic activity while counteracting surgery-related sympathetic inhibition of gastrointestinal motility [21], offering a physiological explanation for the consistently earlier bowel recovery observed in the intervention group.

Consistent with the findings for flatus, the time to appearance of first bowel sounds was significantly shorter among patients who chewed gum (201.5 ± 3.8 vs 280.0 ± 6.3 minutes; p < 0.0001). Time-to-event analysis confirmed this advantage across both surgical approach and gender subgroups. Similar reductions in time to bowel sound recovery have been reported in systematic reviews and trials involving colorectal and upper abdominal procedures [22]. Importantly, this study demonstrated comparable benefits in both open and laparoscopic surgeries, suggesting that the effect of chewing gum is not dependent on surgical invasiveness or the extent of bowel manipulation [23,24].

A statistically significant reduction in postoperative hospital stay was observed in the chewing gum group compared with controls (1.7 ± 1.3 vs 3.1 ± 1.7 days; p < 0.001). However, this finding should be interpreted with caution. The study included a heterogeneous range of abdominal procedures, and postoperative length of stay is influenced by multiple factors beyond gastrointestinal recovery alone. To minimize confounding, perioperative management, including anesthetic technique, analgesic regimen, mobilization protocols, and diet advancement, was standardized across both groups. No differences in postoperative complications or pain management were identified between groups, and no patients experienced wound-related or systemic complications during the observation period. Although the absolute difference in time to gastrointestinal recovery was approximately 100 minutes, earlier restoration of bowel function may have facilitated earlier initiation of oral intake and discharge readiness under institutional criteria. Duration of hospitalization reflects a combination of bowel recovery, symptom control, and overall postoperative stability. The shortened stay observed in this study aligns with prior evidence from Sinz et al. and Atkinson et al., who reported reductions of 0.5-1.5 days associated with gum chewing [25,26]. From a health system perspective, even modest reductions in length of stay may translate into substantial cost savings, particularly in high-volume surgical centers. Nevertheless, the influence of procedural diversity on hospital stay warrants cautious interpretation and should be further explored in procedure-specific studies.

Chewing gum was also associated with a lower incidence and faster resolution of postoperative nausea and vomiting. Symptoms were less frequent at early postoperative time points and resolved significantly sooner in the intervention group (126.0 ± 13.5 vs 237.5 ± 13.1 minutes; p < 0.0001). This effect may be mediated through improved gastric emptying and reduced gastric distension, which in turn decrease vagal afferent stimulation linked to nausea [21]. Prior studies by Liao et al. and Chae et al. similarly demonstrated reductions in postoperative nausea with gum chewing [27,28], and our findings extend this evidence by documenting earlier complete symptom remission.

Multivariable analysis further supported the robustness of these findings, identifying chewing gum as an independent predictor of early gastrointestinal recovery. After adjustment for age and gender, patients in the control group had significantly lower odds of early recovery compared with those in the chewing gum group (OR = 0.29, 95% CI 0.15-0.59; p < 0.001). Neither age nor gender independently influenced recovery outcomes, indicating that the beneficial effects of chewing gum are broadly applicable. Comparable independent associations have been reported in previous analyses of postoperative ileus risk and recovery [29,30].

The absence of adverse events, including wound complications or hemodynamic instability, highlights the safety and tolerability of chewing gum. Its non-pharmacological nature, low cost, and high patient acceptance make it an attractive adjunct to ERAS protocols [6]. Unlike drug-based interventions, chewing gum carries minimal risk and can be easily integrated into routine postoperative care pathways [12].

Limitations

Certain limitations warrant consideration. The single-center design may limit external generalizability to other institutions with differing perioperative practices. Blinding of participants was not feasible due to the nature of the intervention, and no placebo gum was used, which may have introduced performance or expectation bias. Patients aware of receiving the intervention may have been more motivated or optimistic in their recovery, whereas control group participants may have demonstrated lower engagement. Although primary outcomes were objectively assessed by healthcare professionals, the potential influence of behavioral factors cannot be entirely excluded.

The inclusion of both open and laparoscopic abdominal procedures introduces procedural heterogeneity, as these approaches differ in bowel handling and inflammatory response. While randomization ensured a balanced distribution and multivariable analysis did not identify surgical approach as an independent predictor, the study was not powered for detailed procedure-specific subgroup analyses. Furthermore, chewing gum frequency and duration were standardized but not individually titrated, and optimal protocol parameters remain to be determined.

Despite these limitations, the randomized controlled design, consistent perioperative standardization, and robust time-to-event analyses support the internal validity of the findings.

Future directions

Future research should focus on validating the benefits of postoperative chewing gum through multicenter randomized controlled trials with larger and more diverse patient populations to improve generalizability. Although the present study evaluated a broad spectrum of abdominal surgeries to reflect routine clinical practice, further procedure-specific investigations are warranted. In particular, studies stratifying gastrointestinal and non-gastrointestinal surgeries, especially high-risk procedures such as colorectal and pancreatic operations, may help identify subgroups that derive the greatest benefit. Addressing this distinction would provide more granular evidence and strengthen clinical applicability.

Standardization of chewing gum protocols, including timing of initiation, duration, frequency, and type of gum, requires further evaluation to establish evidence-based recommendations. Mechanistic studies incorporating physiological markers such as gastrointestinal hormones, autonomic function indices, and inflammatory mediators could provide deeper insight into the pathways underlying improved bowel recovery. Additionally, integrating cost-effectiveness analyses and patient-reported outcome measures into future trials would support informed clinical decision-making and guide incorporation of chewing gum into ERAS protocols.

## Conclusions

Postoperative chewing gum is a simple, safe, and cost-effective intervention that significantly enhances recovery of gastrointestinal function following abdominal surgery. In this randomized controlled study, chewing gum was associated with an earlier return of bowel activity, a reduced incidence and faster resolution of postoperative nausea and vomiting, and a shorter hospital stay compared with standard care. The consistent benefits observed across surgical approaches and patient subgroups, along with its independent effect demonstrated on multivariable analysis, support the routine incorporation of chewing gum into postoperative care protocols and ERAS pathways to improve patient outcomes and optimize resource utilization.
